# History matters: Preventing severe allergic transfusion reactions

**DOI:** 10.1093/ajcp/aqaf093

**Published:** 2025-08-29

**Authors:** Edina A Wappler-Guzzetta, Asad Shafiq, Umaima Asad, Tushar Chakravarty, Elena G Nedelcu

**Affiliations:** Department of Laboratory Medicine, University of California San Francisco, San Francisco, CA, United States; College of Business and Economics, California State University Fullerton, Fullerton, CA, United States; Centennial High School, Corona, CA, United States; Department of Pathology and Laboratory Medicine, University of California San Francisco, San Francisco, CA, United States; Department of Laboratory Medicine, University of California San Francisco, San Francisco, CA, United States

**Keywords:** allergic transfusion reactions, pretransfusion medications, retrospective study

## Abstract

**Objective:**

Prior studies have shown that pretransfusion medication is not effective in preventing allergic transfusion reactions (ATRs), but these studies did not consider the patient’s history of ATR. This study evaluated whether pretransfusion antiallergy medications decrease the chance of ATRs in patients with a history of severe ATR.

**Methods:**

This single-center, retrospective study investigated the effect of pretransfusion medications on preventing ATRs in patients with a history of at least 1 severe ATR between March 2018 and January 2024. Patient demographics as well as clinical and transfusion reaction data were collected from our electronic health record (EHR) system. Data were analyzed using SPSS (IBM Corp) and machine learning in Python, version 3.12.4.

**Results:**

In our cohort, 53 patients aged 5 weeks to 94 years with 2767 analyzable transfusion encounters had experienced 88 lifelong mild and severe ATRs. Premedication (*P* = .021), regular antiallergy medication (*P* < .001), and washing/volume reduction (*P* = .032) were associated with a statistically significantly lower chance of developing ATRs in our patient population.

**Conclusions:**

Patients with at least 1 severe ATR benefit from pretransfusion administration of antiallergy medications.

**KEY POINTS**

- This study challenges the current paradigm that pretransfusion medication is ineffective in preventing severe ATRs.
- This study confirms that washing and volume reduction decrease ATRs.
- Patients with a history of severe ATRs benefit from pretransfusion antiallergy medications.

## INTRODUCTION

Allergic transfusion reactions (ATRs) are one of the most common adverse events associated with transfusions.^1,2^ Although mild ATRs are primarily associated with blood product wastage and no clinically significant physical harm,^3,4^ patients’ own experience of allergic reactions is often accompanied by considerable distress and anxiety and can even result in refusing to receive transfusions.^5,6^ Furthermore, anaphylaxis is responsible for up to 10% of all transfusion-related fatalities.^7^ For all these reasons, studying severe ATR pathophysiology and therapy, including prophylaxis, is critical.

Numerous prior studies have concluded that pretransfusion medication does not prevent ATRs.^1,5,8^ Pretransfusion medication is therefore not specifically recommended by many transfusion services, including ours. These studies, however, neither evaluated the effect of pretransfusion medications in patients with a history of ATRs nor accounted for regular antiallergy medications. The latter can be common in many patients and confound analysis. Measures such as red blood cell (RBC) or platelet washing or volume reduction and the use of plasma additive solution (PAS) platelets are known to decrease ATR incidence. In many institutes, product washing and volume reduction are used as prophylaxis in patients with a history of severe ATRs,^9,10^ but the protocols for initiating these modifications are institution specific. In line with these thoughts, this study investigated whether pretransfusion medication effectively prevents ATRs in patients with such a history.

## METHODS

### Study design and methods

This study was approved by the Research Ethics Committee of the University of California San Francisco (Institutional Review Board No. 23-39863). It was a retrospective study of patients with severe ATR admitted between 2018 and January 2024 to the University of California San Francisco Medical Center. The list of patients with ATRs during the study period was extracted using SAP Crystal Reports through transfusion reaction sign-outs and billing codes from the electronic health record (EHR) system (Epic Systems Corporation). Diagnosis of ATRs is made according to the current National Healthcare Safety Network–Centers for Disease Control and Prevention (NHSN-CDC) criteria by 4 transfusion medicine attending physicians. Although the transfusion medicine service does not participate in hemovigilance reporting, the system is used for practice standardization using 3 criteria (definition, severity grading, and imputability). Severe ATRs with definite imputability were further selected based on the NHSN-CDC criteria,^11^ where imputability measures how strongly the evidence suggests that the transfusion is directly associated with the ATR. The imputability was definite when no medication or other possible allergen could be associated with the allergic reaction. Only patients who experienced at least 1 severe ATR with definite imputability were analyzed.

### Patient population and data collection

For each patient, the following data were extracted manually from the Epic EHR System into Excel files (Microsoft Excel for Microsoft 365 MSO, version 2405): patient demographics (age, sex), disease or procedures requiring transfusions, allergies, history of transfusion encounters, and detailed data on ATR reactions (eg, clinical symptoms and signs, time of reaction from the start of transfusion, and volume infused). In addition, for all transfusion encounters, we collected data on the presence and type of regular antiallergy medication and premedication, timing, special processing (washing or volume reduction), type of blood product, and whether the patient had had another transfusion within 12 hours. Unreported ATRs were documented in patients’ notes but not reported to the blood bank, and mild ATRs reported for these patients were also included. The term definitions are described in Supplementary material.

### Statistical analysis

Data were analyzed for severe ATR episodes and each transfusion encounter. Descriptive statistics and initial analysis were performed using IBM SPSS, version 20.0, statistical software, with continuous variables presented as mean (SD) and categorical variables expressed as frequencies and percentages. We calculated ATR frequency by dividing the confirmed ATRs by the total number of transfusion encounters (individual transfusion events) for all patients. The potential predictors of severe ATR were product type and processing (RBC washing and platelet volume-reduction only because RBC volume reduction and platelet washing are not performed at our institute), administration of premedication or regular antiallergy medication, and history of transfusion within 12 hours. Data were analyzed using binary logistic regression, and variables with a statistically significant *P* value (*P* < .05) were further included in a multivariable logistic regression model. Post hoc analysis of premedications, such as H1-blockers (diphenhydramine, loratadine, cetirizine), H2-blockers (famotidine), steroids, and epinephrine, as well as antiallergy medications as part of a patient’s regular medication (H1-blockers, H2-blockers, steroids, and epinephrine) and their association with ATR occurrence risk was carried out through logistic regression. Platelet transfusion encounters were analyzed regarding the effect of ABO incompatibility, PAS, and pathogen reduction through binary logistic regression. The goodness of fit of each logistic regression model was evaluated using a likelihood ratio test. Additional analysis was performed in Visual Studio Code using Python, version 3.12.4. Three statistical techniques were assessed: mixed-effects logistic regression, logistic regression, and random forest classifier (RFC). Bivariate analysis of ATR vs risk factors was performed in Visual Studio Code using Python, version 3.12.4, using the χ^2^ statistic for categorical variables and the *t* test for continuous variables; further analysis was conducted using RFC to make the most accurate and robust prediction. For a complete description of statistical methods, refer to Supplementary material.

## RESULTS

### Transfusion reaction rates and severe ATR selection process

A total of 349 558 transfusions were performed during the 71-month study period with 1655 (0.473%) ATRs, for a total rate of approximately 473 per 100 000 transfusions. The most common reaction type was febrile nonhemolytic transfusion reaction at 65.14%, with mild and severe ATRs representing the next 2 most common categories (at 17.4% and 5.26%, respectively). These figures translate to 82 per 100 000 transfusions for mild ATRs and 25 per 100 000 transfusions for severe ATRs. The rest were transfusion-associated circulatory overload in 4.6% patients, transfusion-associated dyspnea in 2.6% of patients, hypotension in 1.9% of patients, acute hemolytic transfusion reactions in 0.4% of patients, transfusion-associated acute lung injuries in 0.24% of patients, septic reactions in 0.06% of patients, and other reactions in 2.4% of patients. A summary of transfusion reaction incidence and rates is provided in Table 1.

**Table 1:** Transfusion Reaction Incidence and Rates

Reaction type	% (No./*n*)	Rate/100 000 transfusions
Febrile nonhemolytic transfusion reaction	65.14 (1078/1655)	308
Mild ATR	17.40 (288/1655)	82
Severe ATR	5.26 (87/1655)	25
Transfusion-associated circulatory overload	4.65 (77/1655)	22
Transfusion-associated dyspnea	2.6 (43/1655)	12
Other	2.42 (40/1655)	11
Hypotension	1.87 (31/1655)	9
Acute hemolytic transfusion reaction	0.36 (6/1655)	2
Transfusion-associated acute lung injury	0.24 (4/1655)	1
Sepsis	0.06 (1/1655)	0.2

Abbreviation: ATR, allergic transfusion reaction; No, number of one type of transfusion reaction; n, number of all transfusion reactions.

**Figure 1:**
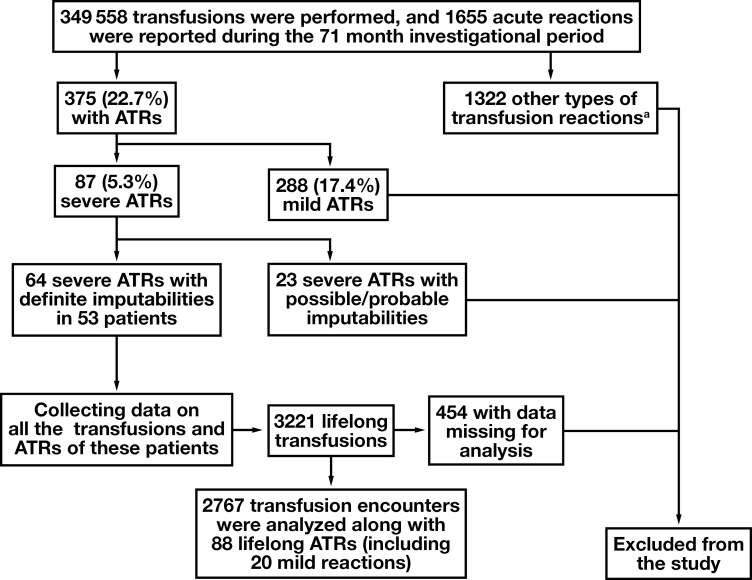
Data collection and study design. To identify severe allergic transfusion reactions (ATRs) with definite imputabilities, we extracted all the acute transfusion reactions from our database for our set time frame; we then selected the ATRs based on their sign-out codes and looked into each sign-out to determine its imputability. We identified 53 patients with 64 severe ATRs in our time frame who had 89 life-long ATRs and 2767 analyzable life-long transfusions. ^a^Febrile nonhemolytic reactions, 65.1%; ATRs, 22.7%; transfusion-associated circulatory overload, 4.6%; transfusion-associated dyspnea, 2.6%; hypotension, 1.9%; acute hemolytic transfusion reactions, 0.4%; transfusion-associated acute lung injuries, 0.24%; septic reactions, 0.06%; and other, 2.4%.

Of the severe ATRs, 53 patients experienced 64 reactions with definitive imputability; reactions with possible or probable imputability were excluded. These 53 patients with at least 1 severe ATR had a total of 3221 transfusion encounters, of which 454 were excluded due to incomplete or missing statistically significant collectible data, leading to 2767 analyzable transfusion encounters (including the ATRs). The elimination of missing encounters in the transfusion-with-no-reaction group was not considered impactful because this group was much larger than the ATR group. Therefore, compensation by other methods or imputation was not deemed necessary. An additional 6 unreported reactions (4 severe and 2 mild ATRs) were identified by a records review in 5 patients; they were included in the total of 88 (20 mild and 68 severe) ATRs. See Figure 1 for a diagram of the patient-selection process. A collection of 2679 transfusion encounters with no reaction served as the control group.

### Patient characteristics, transfusion history, and ATR characteristics

Most patients were female (male to female ratio of 1:1.95). Patient characteristics, transfusion history, and ATR characteristics are summarized in Table 2. The most common diagnosis and transfusion indication was hematologic disease (56%), followed by solid tumors (25%), liver disease and gastrointestinal bleeding (9%), and cardiac (8%) and transplant (2%) surgery. Most (57%) patients had a history of other allergies (eg, drugs, immunoglobulin products, food, insect bites, latex, adhesive tapes), and 26% had multiple allergies. Most patients (44/53 [83%]) had 1 severe ATR, a minority (8/53 [15%]) had 2 severe ATRs, and 1 (2%) patient experienced 4 severe ATRs during the study period. Most (83%) patients, however, experienced a severe ATR as the first ATR (with the patients experiencing either mild or severe ATRs as their first allergic reactions having had transfusions for hematologic diseases), and more than one-third (35%) of these patients had multiple ATRs (range, 2-7 reactions per patient). Overall, the ATR transfusion frequency was 1:36. The ATR transfusion encounters ranged from 1 day to 50 months apart and from 0 to 335 transfusions apart.

**Table 2:** Patient Characteristics, Transfusion History, and Severe ATR Characteristics

Variable	Range/ratio	Mean (SD)
Age	5 wk to 94 y	37.42 (24.3) y
M:F ratio	1:1.95 (18:35)	N/A
Life-long transfusion encounters	1-445/patient	60.7 (77.2)/patient
Time, min^a^	2-160	45.6 (33.2)
Volume, mL^b^	1-1020	222.18 (114.50)

Abbreviations: ATR, allergic transfusion reaction; F, female; M, male.

^a^Time in minutes from the start of transfusion to the time of severe ATR.

^b^Volume in mL of blood product administered at the time of severe ATR.

Clinical symptoms of severe ATR included rash/urticaria (75%), facial swelling (20%), upper (50%) and lower (75%) respiratory symptoms, and gastrointestinal symptoms (1%). Vital signs changes noted in severe ATR were desaturation or hypoxemia, with a mean (SD) oxygen saturation drop of 3.9% (8.5%) (55% of reactions; range, 1%-59% decrease, including 25% of reactions with ≥5% decrease); systolic blood pressure drop of 13.4 (18.6) mm Hg (43% of reactions; range, 1-75 mm Hg, including 28% of reactions with ≥15% decrease); heart rate increase of 13 (17.25)/min (64% of reactions; range, 1-70/min, including 25% of reactions with ≥30% increase); a respiration rate increase of 3.1 (5.9)/min (50% of reactions; range, 1-40/min, including 23% of reactions with ≥5/min increase); and a minor increase in temperature of 0.16 (0.23) °C (range, 0-1 °C), with only 1 patient experiencing a 1 °C increase.

Treatment for severe ATRs included antihistamines (94%), steroids (69%), epinephrine (36%), oxygen (34%), albuterol (26%), norepinephrine or vasopressors (5%), ipratropium (3%), and intravenous fluids (3%); a minority (3%) of patients underwent noninvasive ventilation or intubation (3%).

### Blood product types

Logistic regression was performed to ascertain the effects of premedication, regular antiallergy medication, and washing or volume reduction on the likelihood that participants would have an allergic reaction. The logistic regression model was statistically significant (χ2(4) = 4.713, *P* < .001), and the model correctly classified 96.6% of cases. All independent variables were found to reduce the odds of allergic reactions. The results of the robustness check were consistent with the binary logistic regression results, with no statistically significant differences observed in the magnitude and direction of the hypothesized relationships for any of the variables (product type and processing, premedication, regular allergy medication, and history of transfusion in the past 12 hours).

There was no difference between the product type (RBC vs plasma-rich products) between ATR vs no reaction encounters (*P* = .485), with the following percentages: (1) ATR encounters were platelet (63%), plasma (33%), RBC (2%), and cryoprecipitate (2%), and (2) no reaction encounters were platelet (63%), plasma (29%), RBC (4%), and cryoprecipitate (4%).

Furthermore, ABO-incompatible vs nonincompatible platelet products were not statistically significantly different between the reaction vs no reaction encounters (*P* = .23). About 36% of the platelet transfusions were ABO incompatible in the no-reaction group, with 43% being incompatible among the ATR encounters (including both major and minor ABO incompatibilities). Note that 316 platelet transfusion encounters were excluded from the ABO incompatibility data analysis (not the PAS/pathogen reduction data) because the information was not stored in our EHR system for the year 2018 and before. In addition, PAS vs non-PAS products and pathogen reduction vs non–pathogen reduction platelet products were not statistically significantly different between the ATR and no-reaction encounters (χ2(1) = 1.575, *P* = .209 and χ2(1) = 2.194, *P* = .139, respectively). Notably, 89% of platelets were PAS products; 95% were pathogen reduction.

### The effect of premedication, regular antiallergy medication, and component preparation

The logistic regression model to ascertain the effects of premedication, regular antiallergy medication, transfusion in the past 12 hours, and washing or volume reduction on the likelihood that patients would react was statistically significant (χ2(4) = 4.713, *P* < .001). The model explained 5.3% (Nagelkerke R2) of the variance in the probability of a patient reaction and correctly classified 96.6% of cases. All independent variables were found to negatively affect the odds of having an ATR.

Administration of premedication or a regular antiallergy medication showed a decreased likelihood of developing a reaction (*P* = .021 and *P* < .001, respectively). The odds of a patient who had received premedication having a reaction decreased by 40.2%. Similarly, the odds of a patient developing an ATR who was given regular antiallergy medications beforehand (at least twice before the transfusion, with a regular interval for that specific medication, and was given the last dose ≤12 hours before the transfusion) decreased by 53.5%. Note that regular antiallergy medications were given to these patients as part of treatment for their atopic disease or as part of treatment for their underlying disease.

A transfusion in the past 12 hours was statistically significantly associated with decreased likelihood of a patient reacting. The odds of a patient who had received a transfusion in the past 12 hours having a reaction decreased by 45.8%.

Component processing (RBC washing or platelet volume reduction) statistically significantly lowered the probability of developing an ATR (*P* = .032). However, 9 of the 88 ATRs (2 mild, 7 severe) occurred even with washed RBC/volume-reduced products. A summary of the results of the logistic regression analysis of premedication, regular antiallergy medication, and component preparation variables is provided in Table 3.

**Table 3:** Summary of Logistic Regression Results^a^

Variable	β	SE	Wald χ^2^	*df*	*P* value	Odds ratio	95% CI
Premedication, yes/no	–.514	0.223	5.328	1	.021*	0.598	0.387-0.925
Regular antiallergy medication, yes/no	–.765	0.235	10.569	1	.001**	0.465	0.294-0.738
Other transfusion within 12 h before, yes/no	–.613	0.231	7.011	1	.008**	0.542	0.344-0.852
Washing or volume reduction, yes/no	–.779	0.364	4.583	1	.032*	0.459	0.225-0.936
Constant	–2.352	0.190	152.795	1	<.001***	0.095	N/A

Abbreviations: ATR, allergic transfusion reaction; N/A, not applicable.

^a^The summary of the logistic regression results shows the relationship between the predictors (premedication, regular antiallergy medication, and component preparation variables) and outcome (ATR occurrence). A negative β value shows a decreased likelihood of an encounter being associated with a reaction. An odds ratio <1 indicates that the probability of an event occurring decreases. The administration of premedication, regular antiallergy medication, and washing or volume reduction are associated with a statistically significantly reduced risk of developing an ATR in patients with a history of severe ATRs.

*P < .05. **P < .01. ***P < .001.

The post hoc analysis of premedication revealed a decreased likelihood of a patient developing an ATR with cetirizine (*P* < .001) and famotidine (*P* = .02) but not with diphenhydramine (*P* = .332), loratadine (*P* = .198), or steroids (*P* = .713). Regular antiallergy medication was also statistically associated with decreased ATR risk in the case of antihistamines (*P* <.01) and steroids (*P* = .03) but not with famotidine (*P* = .494). The effect of epinephrine infusion was not assessable due to the lack of infusions during ATR encounters. A summary of the type of medication effect is provided in Table 4.

**Table 4:** Relationship Between Antiallergy Medication Type and Outcome

Variable	β	SE	Wald χ^2^	*df*	*P* value	Odds ratio	95% CI
**Premedication**							
Diphenhydramine	.271	0.279	0.941	1	.332	1.311	0.759-2.264
Loratadine	.803	0.623	1.659	1	.198	2.231	0.658-7.567
Cetirizine	–1.078	0.298	13.115	1	<.001***	0.340	0.190-0.610
Famotidine	–.844	0.358	5.554	1	.018*	0.43	0.213-0.868
Steroids	–.122	0.331	0.135	1	.713	0.885	0.463-1.693
**Regular antiallergy medication**							
Antihistamine^a^	–.768	0.287	7.131	1	.008**	0.464	0.264-0.815
Famotidine	–.240	0.351	0.467	1	.494	0.787	0.396-1.565
Steroids	–.820	0.375	4.778	1	.029*	0.441	0.211-0.919
Constant	–2.617	0.161	264.295	1	.000	0.073	N/A

Abbreviations: ATR, allergic transfusion reaction; N/A, not applicable.

^a^The antihistamine group in regular antiallergy medication included diphenhydramine, loratadine, cetirizine, and fexofenadine. A negative β value shows a decreased likelihood of an encounter being associated with a reaction. An odds ratio >1 (probability of event occurring) signifies that an ATR is likely to occur, while an odds ratio <1 suggests that the ATR probability decreases.

*P < .05. **P < .01. ***P < .001.

The results of the χ^2^ statistic for categorical variables and *t* test for continuous variables developed in Visual Studio Code using Python, version 3.12.4, showed similar results. The blood product type was not statistically associated with the occurrence of ATRs (*P* = .61). Premedication, regular antiallergy medications, component preparation (RBC washing or platelet volume reduction), and different combinations of the drugs were all statistically significant (see Supplementary materials) and consistent with the logistic regression analysis. Transfusion history within 12 hours before the analyzed transfusion episode was negatively correlated with the development of ATRs (*P* <.001), similar to the logistic regression model results. In addition, the use of plasma-rich products was associated with ATRs (*P* <.001), in line with it being the most common transfusion product group for both ATRs and no reaction encounters. Further analysis of the importance of features in the RFC model revealed that the combination of premedication, regular antiallergy medications, and timing of premedication contributed most to the model’s predictions. The receiver operating characteristic curve as a graphical representation of a model’s performance by plotting the true-positive rate against the false-positive rate at various threshold settings revealed improved parameters (accuracy, 94.76%; precision, 95.03%; recall, 94.76%; F1 score, 97%) and improved area under the curve (0.72) after removal of low-importance features, suggesting that the model was reasonably good at distinguishing between positive and negative classes. Additional details of statistical analysis are provided in Supplementary materials.

## Discussion

To our knowledge, this is the first study to show that in patients with a history of severe ATRs, premedication and regular antiallergy medications administered before transfusions reduce the risk of ATRs. The novelty of this study resides in its (1) focus on patients with at least 1 severe ATR occurrence; (2) standardization of ATR diagnosis and selection of definitive imputability; (3) accurate capture of medication data, including routine antiallergy medication administered before transfusion; and (4) robust analysis. Data collection is critical, and in this study, only data from patients with definitive diagnoses and definite imputability were analyzed, eliminating potential confounding factors. Besides focusing on severe ATRs with a transparent and standardized diagnosis algorithm, data collection of medication with possible effects on ATRs, such as antiallergy medications given routinely for reasons other than transfusion, is critical in limiting the confounding effects when investigating ATRs. Taking regular antiallergy medication into account is also important for the known association of ATRs and atopic diseases.^12^ Statistical analysis using logistic regression involved further analysis to reduce bias in parameter estimates due to imbalanced data or small samples and improved robustness checks. The machine learning analysis applied additional Synthetic Minority Oversampling Technique for Nominal and Continuous analysis for the same reason. This approach enhances the performance of classification algorithms on potentially imbalanced datasets by providing a more representative distribution of the minority class.

In our study, ATR rates (107/100 000 transfusions) were lower than those previously reported (232/100 000 transfusions^13^ and 219.5/100 000 transfusions^2^). Regarding the type of products associated with ATRs, prior studies showed that platelets were more likely to cause ATRs than other blood products.^2^

Although there was no difference in blood product types between the ATR and no-reaction encounters in this study, it likely reflects that more platelets were transfused to these patients in general. Furthermore, our study found no difference between PAS and no PAS platelets, even though apheresis platelets in plasma are known to be associated with an increased ATR rate compared with PAS apheresis platelets (760 vs 525/100 000).^13^ The latter products are known to reduce the risk of adverse reactions due to the replacement of 65% of the initial plasma volume with an isotonic solution.^14,15^ The difference between our study and the others is likely due to our very high (89%) inventory of PAS apheresis platelets. This difference also applies to the pathogen-reduction platelets in our study: The 95% pathogen-reduction platelet units used may have contributed to the nonsignificant difference between the ATR and no-reaction encounters when comparing pathogen-reduction and no–pathogen-reduction platelets.

ABO incompatibility was again not statistically significantly different in our study between the reaction and no-reaction encounters. This finding is the opposite of the results of a 2020 article, where the authors found a statistically significant increase in the incidence of ATRs with ABO-incompatible platelet transfusions compared with their ABO-compatible counterparts. Notably, 26% of the ATRs were severe with ABO-incompatible platelet transfusions in that study.^16^ The reason for the difference between our study and the latter one might be the difference in the patient population (ours were all patients with at least 1 severe ATR; theirs was a general population of patients), the high percentage of pathogen-reduction and PAS platelets in our study, and the different percentages of ABO-compatible platelet transfusions in the 2 studies.

In contrast, our study found that washing and volume reduction reduce ATR incidence, in keeping with prior reports.^9,10,17^ Although not unexpected, this finding adds to the knowledge that plasma removal through washing or volume reduction is helpful in patients with severe ATRs, despite the substantial disadvantages associated with component processing (decreasing the expiration time and the effective dose, being time and labor intensive). It is also important to weigh the decision to offer product manipulation because using an open system predisposes blood products to contamination, potentially leading to transfusion-transmitted infection or sepsis. Notably, ATRs may still occur, even with washed or volume-reduced components (approximately 10% in our study).

The question of whether to premedicate has been investigated in numerous prior studies. In the best known randomized prospective trials, the conclusion was that premedication did not reduce the overall ATR rate, but the size of these studies was small^18,19^ in the treatment and placebo groups. Based on these studies, it can be concluded that there is little if any evidence of the effectiveness of antihistamines (specifically, diphenhydramine) on ATRs. These studies did not focus on patients with a history of severe ATRs, however, and information regarding the administration of antiallergy medication was not captured. In addition, the patient-selection process was questionable because the number of patients with hives was lower in the treatment group in 1 study.^20^

In contrast, in the other study, patients with a history of ATRs were excluded, and the only patient experiencing shortness of breath was in the placebo group.^21^ Although another randomized prospective trial investigating the effect of chlorpheniramine maleate found that it prevents delayed (4-24 hours after the transfusion) rash in a reasonably small cohort that did not include severe ATRs,^22^ a retrospective study in pediatric hematology patients showed increased ATR frequency with premedication, likely due to premedication being used more in this category of patients.^23^ Altogether, current recommendations for ATR prophylaxis are based on these studies, which did not investigate premedication effects in patients with a history of severe ATRs. Moreover, the ATR diagnosis was not standardized, and these studies did not address imputability. In addition, no data on the administration of routine antiallergy medication were captured.

Administration of premedication is not unusual in clinical settings before administering a known allergenic agent. For example, 2 doses of steroids are given before infusion of a computed tomography contrast dye.^18^ This finding agrees with our finding that the administration of antihistamines or steroids (at least twice before and within 12 hours of the transfusion) was associated with a decreased risk of developing severe ATRs. Steroids were useful in ATR prevention as regular medications as opposed to premedication, likely due to their more extended time of peak effect and longer half-life. Interestingly, famotidine was not helpful as a regular medication, possibly due to its short half-life. When administered as premedication, famotidine and cetirizine decreased the ATR risk, but diphenhydramine did not. Because there are many differences between the H1-blockers (both between generations and among different drugs),^19^ it is not surprising that their effectiveness varies by situation. As previously mentioned, diphenhydramine is probably a more effective treatment medication than premedication drug in ATRs.^21^

In addition, our study shows that transfusions within 12 hours of another transfusion are associated with a lower risk of developing ATRs (odds ratio = 0.542; *P* <.01), likely due to desensitization. A previous study concluded that in chronically transfused patients, an increased number of transfusions may lead to desensitization.^8^ In our study, however, patients with multiple ATRs experienced their reactions to 335 transfusions approximately 50 months apart. Therefore, the possible desensitization effect must be further investigated to understand its protective timeline better.

Despite its strengths, this retrospective study has several limitations. Although individual data collection was manual and laborious, potential errors cannot be entirely excluded. In addition, this study did not consider the medication dosages or routes and possibly other interventions that might confound the results and reduce the risk of severe ATR over time, such as allergy shots or immunotherapy. Also, potential risks of premedication were not weighted in this study.

Whether blood bank services should indicate premedication and washing or volume reduction to better mitigate ATRs in patients with a history of at least 1 severe ATR requires further studies and the development of optimized protocols, such as those described in other clinical settings.^24,25^

In conclusion, based on this study, product washing or volume reduction and antiallergy medications in the form of premedication or regular medication may benefit patients with a history of at least 1 severe ATR.

## Supplementary Material

aqaf093_suppl_Supplementary_File_1

## Data Availability

Data are available for review.
