# Supplementary material for: History matters: Preventing severe allergic transfusion reactions
Source: Am J Clin Pathol. 2025 Aug 29;164(4):626–33. doi: 10.1093/ajcp/aqaf093 (PMC12495513; doi:10.1093/ajcp/aqaf093)
Supplement: aqaf093_suppl_Supplementary_File_1 [file aqaf093_suppl_supplementary_file_1.pdf]

Supplementary File S1: Complete description of statistical analysis

Data were analyzed for severe ATR episodes, and each transfusion encounter. Descriptive statistics and initial analysis were performed using statistical software (SPSS version 20.0, SPSS Inc., Chicago, IL), with continuous variables being presented as mean ± SD and categorical variables expressed as frequencies. To calculate the frequency of ATRs, the number of confirmed ATRs was divided by the total number of transfusion encounters. The potential predictors of severe ATR were the product type (1=RBC, 2=platelet, 3=plasma, 4=cryoprecipitate), processing such as washing and/or volume-reduction (yes/no), whether the patient was administered premedication (yes/no) or regular allergy medication (yes/no), and history of transfusion in the last 12 hours (yes/no). A binary logistic regression model was used to fit the above-mentioned variables to the odds of having a severe ATR into a stepwise model. Univariate analyses were initially performed, and only the variables with a significant p-value ($p<0.05$) were considered for inclusion in the multivariable logistic regression model. This analysis was followed by a post-hoc analysis of the medications used for pretreatment or as regular medication. For premedication, information about administration of H1-blockers (diphenhydramine, loratadine, or cetirizine), H2-blockers (famotidine), steroids, and epinephrine infusion was collected (no fexofenadine administration was given as premedication); and for anti-allergic drugs as part of patient’s regular medication (H1-blockers, H2-blockers, steroids, and epinephrine) and association with ATR occurrence risk was determined via logistic regression. For regular medications, the types of H1-blockers were not separately analyzed due to the very low frequency of some medications, such as fexofenadine. The platelet transfusion encounters were analyzed separately regarding the effect of plasma additive solution (PAS) and pathogen reduction (PR) via two distinct binary logistic regression models with "reaction encounters" as the dependent variable and "PAS" and "PR" as the independent variables. The goodness-of-fit of each logistic regression model was evaluated using a likelihood ratio test.

Additional analysis was performed using Visual Studio Code using Python 3.12.4. Three statistical techniques were evaluated: mixed effects logistic regression, logistic regression, and Random Forest Classifier (RFC). To address potential dataset imbalance, SMOTENC (Synthetic Minority Over-sampling Technique for Nominal and Continuous data) was performed to generate synthetic samples by interpolating between existing minority class samples for continuous features, ensuring that the categorical features remain within their possible values. Further bivariate analysis of ATR vs. risk factors was performed in Visual Studio Code using Python 3.12.4. by Chi-Square statistic for categorical variables and *t*-test for continuous variables. Only variables with a significant P -value ($P < .05$) were considered for further analyses by RFC to make a more accurate and robust prediction.
